# Sleep Deprivation Influences Circadian Gene Expression in the Lateral Habenula

**DOI:** 10.1155/2016/7919534

**Published:** 2016-06-19

**Authors:** Beilin Zhang, Yanxia Gao, Yang Li, Jing Yang, Hua Zhao

**Affiliations:** ^1^Department of Physiology, College of Basic Medical Sciences, Jilin University, Changchun 130021, China; ^2^Neuroscience Research Center, First Hospital of Jilin University, Changchun 130021, China

## Abstract

Sleep is governed by homeostasis and the circadian clock. Clock genes play an important role in the generation and maintenance of circadian rhythms but are also involved in regulating sleep homeostasis. The lateral habenular nucleus (LHb) has been implicated in sleep-wake regulation, since LHb gene expression demonstrates circadian oscillation characteristics. This study focuses on the participation of LHb clock genes in regulating sleep homeostasis, as the nature of their involvement is unclear. In this study, we observed changes in sleep pattern following sleep deprivation in LHb-lesioned rats using EEG recording techniques. And then the changes of clock gene expression (Per1, Per2, and Bmal1) in the LHb after 6 hours of sleep deprivation were detected by using real-time quantitative PCR (qPCR). We found that sleep deprivation increased the length of Non-Rapid Eye Movement Sleep (NREMS) and decreased wakefulness. LHb-lesioning decreased the amplitude of reduced wake time and increased NREMS following sleep deprivation in rats. qPCR results demonstrated that Per2 expression was elevated after sleep deprivation, while the other two genes were unaffected. Following sleep recovery, Per2 expression was comparable to the control group. This study provides the basis for further research on the role of LHb Per2 gene in the regulation of sleep homeostasis.

## 1. Introduction

Clock genes are a class of molecules essential for the generation and maintenance of circadian rhythms. The protein products of clock genes collaborate to establish a network of autoregulatory feedback loops. Such loops are thought to be exclusively involved in the maintenance of a ~24 h oscillation cycle and the regulation of physiological functions. The core loop includes a brain and muscle ARNT-like protein 1/circadian locomotor output cycles kaput (Bmal1/clock) heterodimer that binds to Enhancer Box (E-box) containing elements on the promoters of the core clock genes, period (Per1, Per2, Per3), and cryptochrome (Cry1, Cry2) [1, 2]. Knockout or mutation of circadian genes can cause an alteration in rhythm, such as in Bmal1 knockout mice, which demonstrate loss of activity-based rhythmicity [3]. Similarly, the absence of Per [4–6] or Cry gene [7] expression in mice can result in a shorter circadian period. Furthermore, transgenic clock gene-deficient mice demonstrate robust circadian rhythms when compared to wild-type animals [8]. These studies suggest that clock genes play an important role in the generation and maintenance of circadian rhythms in mammals.

Sleep is one of the most renown functions influenced by circadian rhythms and is modulated by both the circadian clock and sleep homeostasis [9, 10]. The influence of genes associated with the circadian clock on sleep pattern and duration has been well characterized in recent years. For example, the knockout of NPAS2 in mice produces a reduction in Non-Rapid Eye Movement Sleep (NREMS) and decreased NREMS delta power following sleep deprivation [11–13]. Knockout of Cry1/2 reportedly decreased the number of awakenings and increased NREMS delta power during the first hour after sleep deprivation [14]. The Per gene mutation mice was observed to have increases in wakefulness time. The NREMS delta power was enhanced in the Per gene mutation mice after sleep deprivation, compared with wide-type mice [15, 16]. Additionally, both Per1 and Per2 levels in cerebral cortex and forebrain were increased and Dpb mRNAs were decreased by using qPCR after enforced waking and these reverted to control levels within 2 h of recovery sleep [17, 18]. However, the mRNA levels of Cry, Bmal1, and other clock genes demonstrated no significant changes following sleep deprivation and recovery time [18]. In situ hybridization showed that sleep deprivation enhanced Per1 expression in the forebrain, cerebral cortex, cerebellum [17], striatum, and olfactory bulb [14]. These studies suggest that Per expression is likely dependent on the region's involvement in sleep homeostasis.

A primary region implicated in circadian rhythm regulation and sleep homeostasis is the habenula (Hb). The Hb features a semiautonomous circadian oscillator localized to its lateral region, the LHb. Circadian oscillators reportedly have the independent capacity to generate circadian rhythms independently of the suprachiasmatic nucleus (SCN) pacemaker [19, 20]. The LHb in particular has been linked to the production of circadian functions due to vasopressinergic connections with the SCN, which conveys circadian information to relevant regions [21]. Additionally, the LHb has been reported to generate circadian firing-rate rhythms for at least two cycles in vitro [19] and to initiate circadian rhythms of clock gene expression [22]. It is reported that electrical stimulation of the LHb increases NREMS in cats [23]. These results suggest a role for the LHb in the regulation of sleep homeostasis and circadian rhythms. However, the relative involvement of clock gene expression in regulation of sleep homeostasis is still unknown. In the current study, we examined the effect of LHb lesions on the sleep rebound induced by a 6 h sleep deprivation. And then we further detected the changes of clock gene (Per1, Per2, and Bmal1) expression in the LHb after 6-hour sleep deprivation using qPCR.

## 2. Materials and Methods

### 2.1. Animals and EEG Recording

Six male Wistar rats (210–230 g) obtained from the Center for Experimental Animals, Jilin University (Certification number SCXK (Ji) 2007-0003), were surgically prepared for EEG and electromyographic (EMG) recording. Rats were maintained under a 12 h light-dark cycle (lights on at 08:00 am), while under anesthesia with 10% chloral hydrate (0.35 mL/100 g), stainless steel jewelry screws were implanted into regions of the skull overlying the frontal and parietal cortices for EEG recording. Two stainless steel electrodes were placed into the dorsal neck muscles to record EMG data. In addition, a guide cannula was stereotaxically implanted above the LHb (coordinates: posterior 3.6 mm, lateral 0.6 mm, ventral 4.2 mm from bregma and dura, Paxinos and Watson, 1986). The guide cannula was fixed onto the skull with dental cement. All electrodes were placed in a six-pin plastic plug and secured onto the skull using dental cement. In the study, the difference between sleep and wake cycle following 6 h sleep deprivation before and after LHb lesions in the same group rats was analyzed to determine the effect of LHb lesions on the sleep rebound induced by a 6 h sleep deprivation. After allowing 7 days for surgical recovery, the first time of EEG recording for 24 hours was performed at 14:00 pm. The second time of EEG recording followed by a 6 h period of sleep deprivation starting at 14:00 pm was performed for 18 h, starting at 20:00 pm and ending at the second day at 14:00 pm. We repeated the same experiment procedure as mentioned above seven days after LHb lesions in the same group of rats. Sleep deprivation in all experiments was achieved by gentle handling.

In order to damage the LHb, 0.2 *μ*L kainic acid (1.5 *μ*g/*μ*L) was slowly injected to the LHb (over 2 min) using a needle connected with a guide cannula, its tip extended 0.5 mm below the cannula end implanted.

The recording system consisted of a computer with an analog-to-digital converter (6036E, NI company) and a Grass Model 12 Neurodata Amplifier system (Grass Instrument Division of Astro-Med, Inc., West Warwick, RI). The signals were amplified by 12A5 amplifiers and acquired by a computer program (gammar, Grass Instrument Division of Astro-Med, Inc., West Warwick, RI). The EEG data recordings were scored manually by Sleepsign software (KISSEI Nagano, Japan) in 10 s epochs for wakefulness, NREMS, and Rapid Eye Movement Sleep (REMS) according to the standard criteria [24]. The wake state was identified by the presence of desynchronized EEG (6–30 Hz) and high EMG activity. A high-amplitude slow wave (0.75–4 Hz) together with a low EMG tone relative to awake is NREM sleep. REM sleep consisted of regular *θ* activity (6–10 Hz) coupled with low EMG relative to NREM sleep. The typical EEG patterns were seen in Figure 2(a).

### 2.2. qPCR Study

In this part, 128 male Wistar rats (190–210 g) were maintained in 12 h light-dark cycles (light on at 7:00 am) with food and water available ad libitum prior to experimentation. Rats were divided into four groups: a sleep deprivation group, control for sleep deprivation group, recovery group, and a control for recovery group. Drawing from the initial sleep deprivation experiment, rats were sleep-deprived for 6 h starting at 7:00 am by gentle handling and sacrificed at 1:00 pm together with non-sleep-deprived controls. The recovery group was allowed 2 h of recovery sleep following 6 h of sleep deprivation and sacrificed at 3:00 pm together with non-sleep-deprived controls.

The brains were rapidly removed, and two 500 *μ*m thick coronal slices containing LHb were collected by vibration cutting machine (ZQP-86, Shanghai Five-Phase Instrument Co., Ltd., Shanghai, China) from Bregma −3.12–−4.20 mm. The medial habenula consists mostly of dense cells, and lateral habenula consists of more loosely arranged cells [25]. The boundary between medial and lateral habenula is visualized. We dissected the LHb tissue with needle along the outer edge of the boundary to avoid the pollution of the medial habenular tissue. However, it is difficult to extract selectively lateral part of the LHb because there were no obvious boundaries visualized between lateral and medial parts of the LHb. Thus the complete LHb was targeted in the study.

The LHb tissue from 3 rats was put into one Eppendorf (EP) tube as one sample. There are 24 rats in each group; therefore, each group had 8 samples, which were frozen at −80°C. The samples of rat LHb were homogenized using TRIzol Reagent (Invitrogen) and then separated into three phases by chloroform: a lower red, phenol-chloroform phase, an interphase, and a colorless upper aqueous phase. RNA exists exclusively in the aqueous phase. After precipitating the RNA from the aqueous phase by mixing with isopropyl alcohol (Beijing Chemical Works, Beijing, China), the RNA pellet was washed with 75% ethanol (Beijing Chemical Works, Beijing, China) and dried at room temperature. Finally, RNA pellets were redissolved in RNase-free double distilled water. The concentration as well as purity of RNA was detected by a microplate reader (Biotech Company) to achieve RNA quantification.

qPCR was performed using SYBR® Premix Ex Taq II 2x (Tli RNaseH Plus, TAKARA). Prior to cDNA synthesis, RNA samples were treated with gDNA eraser (TAKARA ambion) to remove genomic DNA contamination, and 400 ng of total RNA was converted into first strand cDNA with Oligo(dT) and Random 6 mers (TAKARA ambion). Primers for three circadian genes (Per1, Per2, and Bmal1, Table 1) were designed and synthesized by Sangon Biotech Company (Shanghai, China) and checked by primer-BLAST at NCBI. Quantitative fluorescence PCR was performed using an Mx3000P qPCR System. This incorporated several steps: 95°C denaturation for 30 s, 40 cycles of 95°C denaturation for 5 s, and annealing and extension at 60/63°C for 20 s (Table 1). Data were analysed by MxPro QPCR software. Ct value for Per1, Per2, and Bmal1 was normalized to *β*-actin Ct value. The relative expression level of each clock gene sample was calculated by the 2^−ΔΔCt^ method. The mean and SEM of relative expression level were analyzed.

### 2.3. Histology Examination

**Figure 1:**
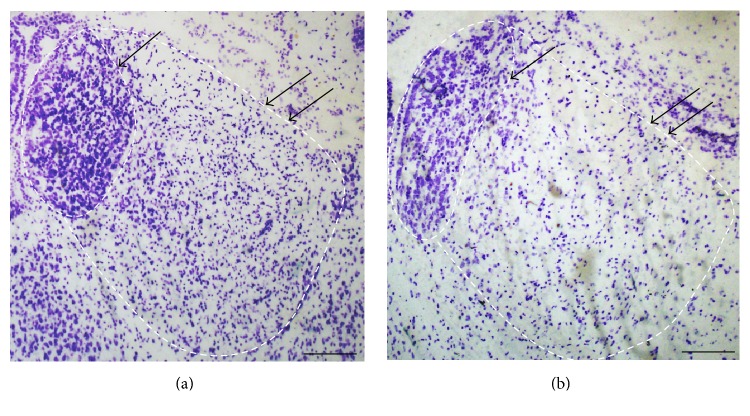
A crystal violet staining photomicrograph of LHb lesion. (a) showed the normal Hb and (b) showed the lesion of LHb. The single arrow identifies the MHb, whereas the double arrows identify the LHb. Scale bars represent 100 *μ*m.

Histological staining with Cresyl Violet acetate was used to evaluate extent of the LHb lesion. Nylon body, a characteristic structure of the neurons, could be stained by Cresyl Violet acetate. The areas of the lesion were not stained with Cresyl Violet acetate (1% Cresyl Violet acetate in PBS, pH = 3.8) because of the loss of Nylon body. Therefore, we can estimate the range of lesion based on the staining under microscope (Figure 1). The data of damaged area outside of the LHb are not included in the statistics.

### 2.4. Statistics

Data from 18 h EEG recording which follow sleep deprivation before and after the LHb lesion were analyzed by paired *t*-test (SPSS11.0 software) and presented as means ± SEM. EEG data plotted per 6 h for a 24 h period following deprivation before and after lesion were assessed by repeated measures one-way analysis of variance (ANOVA), with two-way ANOVA process to achieve two comparisons between sleep and wake times before and after sleep deprivation at each time point. Unpaired *t*-tests were used to analyze qPCR results.

## 3. Results

### 3.1. Effects of LHb Lesion on the Sleep-Wake Cycle in Rats following Sleep Deprivation

**Figure 2:**
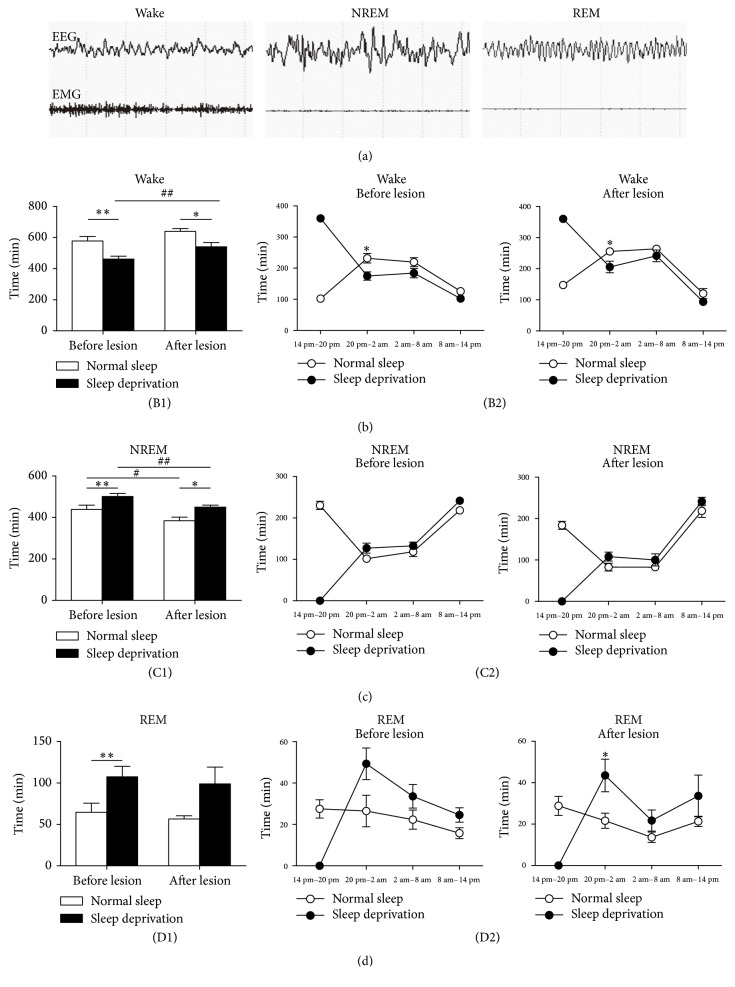
The effect of LHb lesion on sleep-wake times following sleep deprivation in rats. (a) indicates the typical EEG and EMG recording, respectively, for wake, NREMS, and REMS times. The wake state was identified by the presence of desynchronized EEG and high EMG activity. The NREMS consisted of high-amplitude slow waves together with a low EMG relative to awake. The REMS was identified by the presence of regular *θ* activity coupled with low EMG relative to NREMS. (B1), (C1), and (D1), respectively, show the wake, NREMS, and REMS times following sleep deprivation before and after LHb lesion. Data from 18 h EEG recording were analyzed and presented as means ± SEM (*n* = 6). ^*∗*^ *P* < 0.05, ^*∗∗*^ *P* < 0.01, compared, respectively, difference between the wake, NREMS, and REMS times before and after sleep deprivation. ^#^ *P* < 0.05, ^##^ *P* < 0.01, compared, respectively, difference between the wake and NREMS times following deprivation before and after LHb lesion. (B2), (C2), and (D2) show, respectively, the graphs of wake, NREMS, and REMS plotted per 6 h for a 24 h period before and after lesion. ^*∗*^ *P* < 0.05 compared with sleep deprivation at the same time point.

In this study, we compared the difference between sleep and wake cycle following 6 h sleep deprivation before and after LHb lesions in the same group rats to determine the effect of LHb lesions on the sleep rebound induced by a 6 h sleep deprivation. The first period of sleep deprivation was initiated prior to lesioning, wherein the mean wake time significantly decreased from 577.31 ± 29.67 min to 461.14 ± 18.15 min (*t* = 6.459, df = 5, *P* = 0.0013, Figure 2(b)(B1)). Additionally, the duration of NREMS was increased significantly from 438.17 ± 21.2 to 501.36 ± 14.23 min (*t* = 5.102, df = 5, *P* = 0.0038, Figure 2(c)(C1)). The duration of REMS also increased (*t* = 4.602, df = 5, *P* = 0.0058, Figure 2(d)(D1)). Therefore, the initial experimental phase demonstrated that sleep deprivation could elicit a sleep rebound effect in rats. The second period of sleep deprivation was performed 7 days after lesioning of the LHb. At this stage, the wake time was significantly reduced from 639.31 ± 18.22 min to 540.44 ± 27.32 min (*t* = 3.788, df = 5, *P* = 0.0128, Figure 2(b)(B1)), and NREMS was significantly increased from 384.14 ± 17.22 min to 449.58 ± 9.39 min (*t* = 3.204, df = 5, *P* = 0.0239, Figure 2(c)(C1)). Further analysis demonstrated that the duration of wakefulness following the sleep deprivation was longer after LHb lesion than before LHb lesion (540.44 ± 66.91 min versus 461.14 ± 44.46 min, *t* = 5.787, df = 5, *P* = 0.0022, Figure 2(b)(B1)) and NREMS was shorter after LHb lesion than before LHb lesion (439.69 ± 31.29 min versus 493.14 ± 51.07 min, *t* = 4.335, df = 5, *P* = 0.0075, Figure 2(c)(C1)). Figures 2(b)(B2), 2(c)(C2), and 2(d)(D2) showed sleep-stage distributions following 6 h sleep deprivation before and after LHb lesion. In the first 6 h after SD, the LHb lesion decreased the amplitude of increased REMS and reduced wake time by sleep deprivation. No significantly difference was found between sleep and wake times before and after microinjected 0.2 *μ*L vehicle to the LHb in sham surgery group.

### 3.2. Effects of Sleep Deprivation on Circadian Gene Expression in the LHb

**Figure 3:**
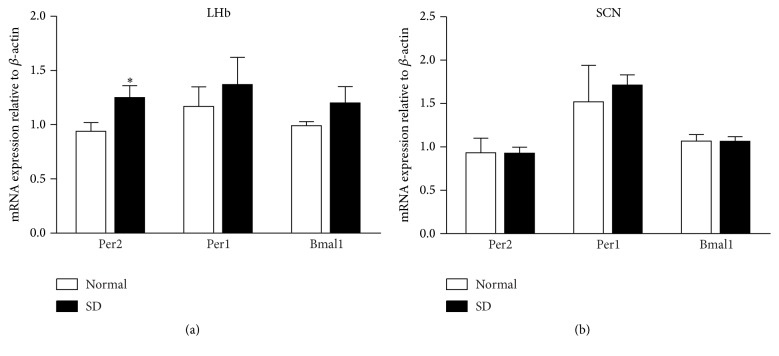
Sleep deprivation alters mRNA levels of clock genes. qPCR analysis of the expression of three clock genes (Per1, Per2, and Bmal1) in the LHb (a) and SCN (b) of the rat. Data was presented as mean ± SEM (*n* = 8/each group). ^*∗*^ *P* < 0.05, compared with control group (by unpaired *t*-test).

The mRNA levels for Per1, Per2, and Bmal1 were quantified using qPCR for two brain regions, the SCN and LHb. The mRNA levels of Per2 in the LHb increased following 6 h of sleep deprivation, wherein Per2 expression in the sleep deprivation group (1.25 ± 0.11, *n* = 8) was significantly higher than control group (0.94 ± 0.08, *n* = 8; *t* = 2.326; df = 14; *P* < 0.05). Comparatively, sleep deprivation did not affect Per1 or Bmal1 expression in the LHb (Figure 3(a)). The mRNA levels of these three genes in the SCN showed no alterations after 6 h of sleep deprivation (*n* = 8, Figure 3(b)).

### 3.3. Circadian Gene Expression Changes following 2 h Recovery Sleep

**Figure 4:**
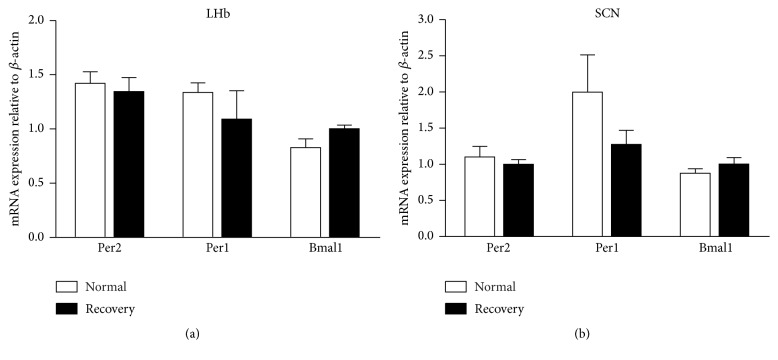
qPCR analysis of Per1, Per2, and Bmal1 levels in the LHb following 2 hours of recovery after sleep deprivation. Data was presented as mean ± SEM (*n* = 8/each group).

Per1, Per2, and Bmal1 mRNA levels were not affected following 2 hours of recovery after sleep deprivation in either the LHb or SCN, compared with the control group (*n* = 8/each group, Figures 4(a) and 4(b)). No significant differences were observed in the LHb for Per1 expression for the recovery group (1.09 ± 0.26) when compared with the control group (1.34 ± 0.09, *t* = −0.896, *P* > 0.05). A similar pattern was observed for Bmal1 expression between the control group (0.83 ± 0.08) and the recovery group (1.00 ± 0.03, *t* = 2, *P* > 0.05). Likewise, there was no difference in Per2 expression between the control group (1.42 ± 0.11) and the recovery group (1.35 ± 0.13, *t* = −0.44, *P* > 0.05). A similar pattern was observed for these genes in the SCN.

## 4. Discussion

Sleep deprivation tends to produce a correlated increase in sleep rebound, an effect that was demonstrated in the current study through an increased duration of both NREM and REM sleep. This suggests that sleep homeostasis may play an important role in the regulation of sleep pattern [9, 26]. In this experiment, we determined the effect of LHb lesions on the sleep rebound induced by 6 h sleep deprivation. Compared to prelesion data, the postlesion sleep recovery time induced by sleep deprivation was reduced as shown by a decrease in NREMS and an increase in wake time. When the difference between sleep and wake time before and after LHb lesions in the same group rats was analyzed, we found that the LHb lesion could induce a decrease in NREMS time, which is consistent with the results of Goldstein's studies, showing that stimulation the LHb could increase NREMS time [23]. It is considered that the role of the LHb lesion on sleep rebound evoked by sleep deprivation may relate to its role in promoting sleep. Previous studies have also suggested that the LHb has a close functional connection with several structures implicated in sleep homeostasis, including the preoptic hypothalamus, the raphe nuclei, and the pineal gland [27–29]. The LHb also regulates the release of various neurotransmitters linked to homeostatic regulation, such as adenosine, melatonin, and monoamine neurotransmitters [30–32]. These studies provide further evidence to suggest a role for the LHb in the regulation of sleep homeostasis.

The sleep-wake cycle features its own distinct circadian rhythm, which contributes to maintenance of sleep-wake cycle [10, 33]. It has been well-established that clock genes are not only the basis of the generation of circadian rhythm, but also related to the regulation of sleep homeostasis [34, 35]. They were reported to have an increase in the NREMS delta power in the Per gene mutation mice after sleep deprivation, compared with wide-type mice [15, 16]. Additional study showed that the Per gene induction was found in the basal forebrain and cerebral cortex of mice following sleep deprivation [17, 18]. The results of the current study showed that sleep deprivation induces increase of Per2 expression in the LHb, whereas the increase returns to basal levels following sleep recovery. Drawing from the data obtained in the current study, sleep not only is affected by the activity of clock genes, but is also able to regulate their expression. This suggests that clock genes are involved in the regulation of sleep homeostasis.

There is a link between clock gene expression and sleep-related processes; however the underlying regions supporting sleep homeostasis have received relatively limited focus. Numerous studies have provided evidence to suggest the importance of the SCN as the mammalian circadian pacemaker, but in recent years, increasing studies have demonstrated a growing role for the LHb, which is thought to contribute significantly to the production of higher brain functions and may be implicated in the pathology of neurological disease [32, 36–39]. The LHb is considered to feature a semiautonomous circadian oscillator, similar to those identified in the SCN, retina, and olfactory bulb [20]. As a function of this, the ability of the LHb to influence circadian rhythm production and maintenance has garnered interest in recent years. The LHb neurons have been shown to have rhythmic electrical activity [19], whereas Per2 mRNA and protein levels appear to oscillate with circadian characteristics [22, 40]. This study supports an additional role for the LHb in sleep homeostasis, in which lesions to the LHb suppress increases in sleep rebound time and enhance wakefulness following sleep deprivation.

Despite evidence of Per2 induction in the LHb, we found no change in the expression of Per2 in the SCN after sleep deprivation, which was consistent with the experimental results of a similar study [41]. Sleep deprivation is a complex stimulus that invokes arousal and induces mild stress. Further to this, previous studies have reported a link between Per expression and the pattern and duration of stress [42]. Since the LHb has demonstrated involvement in the production of stress responses [32, 43], the alteration of Per2 expression observed in the current experiment may be linked to stress following exposure to sleep deprivation. As of yet, no studies have reported the involvement of the SCN in the production of stress, which might explain the absence of Per2 gene induction in the SCN following sleep deprivation.

Our findings showed that LHb lesion significantly shortens the sleep rebound time induced by sleep deprivation, suggesting an important role for the LHb in sleep homeostasis. Furthermore, Per2 expression in the LHb is significantly increased following sleep deprivation, indicating that the action of Per2 in the LHb may be related to the regulation of sleep homeostasis. However, further research is needed to prove that.

## Figures and Tables

**Table 1:** RT-qPCR primers used for the analysis of clock gene expression.

Gene	Primers (5′ to 3′)	Temperature (°C)
Per1	Forward (5′-3′): gaggagccagagaggaaagagt Reverse (5′-3′): caggaagaggaggcacatttac	63
Per2	Forward (5′-3′): agcaacaccacctttcacaa Reverse (5′-3′): cgtaggcttagaccaccatc	63
Bmal1	Forward (5′-3′): caacccatacacagaagcaaac Reverse (5′-3′): acagattcggagacaaagagga	60
*β*-actin	Forward (5′-3′): agccatgtacgtagccatcc Reverse (5′-3′): ctctcagctgtggtggtgaa	60/63
